# A rare case of mosaic partial tetrasomy 18p presenting with oligomenorrhea and intellectual disability: a case report

**DOI:** 10.3389/fmed.2026.1757421

**Published:** 2026-01-22

**Authors:** Guosheng Deng, Weiwu Liu, Yuqing Lai, Jinjie Pan, Yanyan Chen, Jujie Song, Donghua Zhang, Xiafei Liang, Sisi Ning, Yinghong Lu, Yunning Liang, Derong Li

**Affiliations:** 1 Clinical Laboratory, Yulin Maternal and Child Health Care Hospital, Yulin, Guangxi, China; 2 Obstetrics Outpatient Clinic, Yulin Maternal and Child Healthcare Hospital, Yulin, Guangxi, China; 3 Surgical Department, Yulin Maternal and Child Health Care Hospital, Yulin, Guangxi, China; 4 Reproductive Medicine Center, Yulin Maternal and Child Health Care Hospital, Yulin, Guangxi, China

**Keywords:** chromosome, chromosome abnormality, CNV-seq, oligomenorrhea, tetrasomy 18p

## Abstract

**Background:**

Tetrasomy 18p syndrome is an extremely rare chromosomal disorder that is often the result of an additional isochromosome for the short arm of chromosome 18. Most cases result from *de novo* mutations, although familial cases have also been reported. In this article, we report for the first time a case of a 17-year-old female patient with mosaic tetrasomy 18p.

**Objective:**

This study aimed to investigate the genetic and clinical characteristics of patients with tetrasomy 18p and to enhance the understanding of tetrasomy 18p.

**Methods:**

A female patient presenting with oligomenorrhea, distinctive physical features, and intellectual disability underwent chromosomal karyotype analysis via G-, C-, and N-banding techniques. Upon the detection of abnormal results, high-throughput genomic copy number variation sequencing (CNV-seq) was performed to establish a definitive diagnosis. Additionally, chromosomal karyotype analysis was conducted on both parents and the patient’s three younger sisters.

**Results:**

The G-banding karyotype results indicated 47,XX,+mar[85]/46,XX[15], suggesting a mosaic condition with one additional marker chromosome. C-banding revealed one centromere on the small marker chromosome, whereas N-banding revealed no satellite. CNV-seq analysis revealed a 14.86 Mb mosaic duplication spanning 18p11.32-p11.21, with an estimated copy number of 3.5. On the basis of these findings, the final chromosomal karyotype was designated 47,XX,+i(18)(p10)[85]/46,XX[15]. No chromosomal abnormalities were identified in the karyotypes of the patient’s parents or three sisters, indicating that this is a *de novo* mutation.

**Conclusion:**

The patient’s oligomenorrhea, distinctive features, and intellectual disability may be associated with a partial duplication of the short arm of chromosome 18. Previous reports on patients with tetrasomy 18p have focused primarily on preschool or prepubescent patients. Here, we present the first reported case of a 17-year-old female with mosaic tetrasomy 18p. The absence of fingerprints on the palms and oligomenorrhea may represent novel characteristics of this condition. For such rare chromosomal abnormalities, the combined use of G-banding, C-banding karyotype analysis, CNV-seq technology, and comprehensive clinical evaluation can facilitate early diagnosis, genetic counseling, and personalized treatment.

## 1 Introduction

Tetrasomy 18p results from an abnormal extra chromosome composed of two copies of the short arm of chromosome 18, creating an isochromosome 18p that is present in each cell. It is an extremely rare chromosomal aberration disease, with an incidence of approximately 1/140,000 to 1/180,000 (1, 2). Typical clinical manifestations of tetrasomy 18p include growth retardation, intellectual disability, and cognitive impairment; dystonia; distinctive facial features; hearing and vision abnormalities in some patients; congenital heart disease; renal dysplasia; epilepsy; short stature; spinal column abnormalities; mental abnormalities; and other clinical manifestations. In this study, we employed chromosomal karyotype analysis and high-throughput copy number variation sequencing (CNV-seq) to perform genetic testing on a patient presenting with oligomenorrhea accompanied by distinctive facial features and intellectual disability. The diagnostic results identified the case as a mosaic form of partial tetrasomy 18p syndrome, with a karyotype of 47,XX,+i(18)(p10)[85]/46,XX[15]. We further investigated the correlation between the clinical manifestations and genetic characteristics to provide scientific evidence for clinical diagnosis and genetic counseling.

### 1.1 Case presentation

A 17-year-old female patient, 150 cm tall, presented to our hospital in January 2025 for evaluation of irregular menstruation over the past 5 years. The patient experienced menarche at age 12, with subsequent oligomenorrhea-manifesting as only three menstrual cycles between ages 12 and 17. She has a low hairline, strabismus, small and low-set ears, an absence of fingerprints, curved index fingers, and intellectual disability. Although she was capable of basic communication, performing household chores, and maintaining personal hygiene, the patient exhibited functional limitations such as illiteracy, inability to write, and impaired calculation skills. The patient’s parents exhibited normal phenotypes and were not consanguineous. The maternal uncle had an intellectual disability, whereas the patient’s three younger sisters presented no abnormalities. Initial evaluation included hormonal assays, chromosomal analysis, and gynecological ultrasound examination sound. Gynecological ultrasound revealed a uterine size of approximately 33*31*17 mm. The hormonal findings were as follows: follicle-stimulating hormone at 5.61 mIU/mL (normal value, 1.38–5.47 mIU/mL), testosterone at 0.38 ng/mL (normal value, 0.11–0.57 ng/mL), luteinizing hormone at 9.22 mIU/mL (normal value, 0.56–14 mIU/mL), prolactin at 7.25 ng/mL (normal value, 5.18–26.53 ng/mL), estradiol at 43 pg/mL (normal value, 21–312 pg/mL), and anti-Müllerian hormone (AMH): 4.75 ng/mL (normal value, 0.77–14.15). In February 2025, further CNV-sep testing was performed due to the detection of chromosomal abnormalities. Following karyotype and CNV-seq analysis, the patient was diagnosed with 47,XX,+i(18)(p10)[85]/46,XX[15] in March 2025. The patient’s parents, concerned about their daughter’s irregular menstrual cycles and the potential impact on her health and fertility, requested medical treatment with the expectation of restoring regular menstruation. Treatment with progesterone capsules (100 mg twice daily) was initiated to induce menstruation, with unsatisfactory results. From June 2025, the patient received Complex Packing Estradiol Tablets/Estradiol and Dydrogesterone Tablets 1 mg once daily combined with progesterone capsules (100 mg twice daily), which resulted in regular menstrual cycles. The patient provided written informed consent. This study was approved by the Ethics Committee of Yulin Maternal and Child Health Care Hospital.

## 2 Methods

### 2.1 Karyotype

One milliliter of the patient’s blood was inoculated into a bottle containing human peripheral blood lymphocyte culture medium (Guangzhou Baidi Biomedical Co., Ltd.) and incubated at 37 °C for 72 h. Subsequently, 20 μL of colchicine (20 μg/mL) was added, and the cells were harvested after 3 h. Routine slide preparation, including G-banding, C-banding, and N-banding, was then performed. Karyotyping was conducted via a fully automated scanning microscope and image analysis system (Leica, United States) for automatic scanning and identification. The data were automatically transmitted to AutoVision® Chromosome Intelligent AI Analysis Software (*De Novo* Biotechnology Co., Ltd., Hangzhou, China). For mosaic analysis, 100 cells were counted, and five karyotypes were analyzed. Karyotype descriptions adhered to the ISCN 2020 guidelines.

### 2.2 Copy number variation sequencing (CNV-seq)

DNA was extracted from the samples via a commercial kit (Berry Genomics, Beijing, China) and compared with genomic databases to calculate coverage depth values for chromosomal abnormality analysis. The clinical significance of copy number variations (CNVs) was classified into five categories—pathogenic, likely pathogenic, variant of uncertain significance, likely benign, and benign—according to ACMG guidelines.

## 3 Results

**Figure 1:**
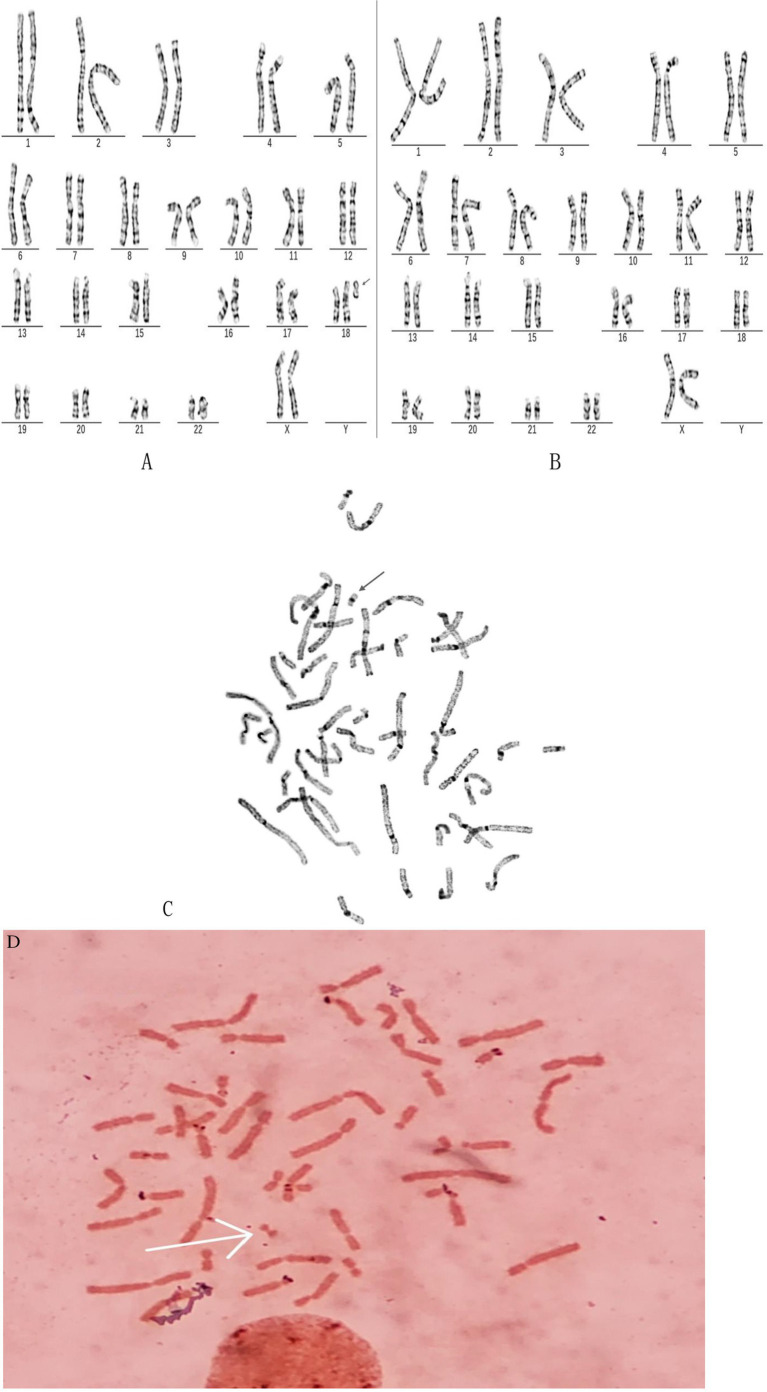
Karyotype of patients with 47,XX,+i(18)(p10)[85]/46,XX[15]. **(A)** 47,XX,+i(18)(p10)[85]: G-banding chromosomal analysis showing isochromosome 18p (arrow). **(B)** 46,XX[15]. **(C)** The C-banding results demonstrated that the small marker chromosome contained a single centromere. **(D)** The N-banding results revealed no satellite structure.

**Figure 2:**
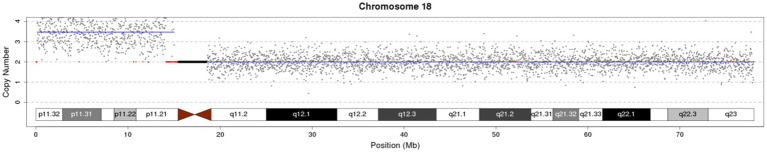
The results of CNV-seq.

Chromosome karyotype analysis revealed G-banding results of 47,XX,+mar[85]/46,XX[15], indicating the presence of chimerism with one additional marker chromosome (Figures 1A,B). C-banding revealed that the small marker chromosome contained a single centromere (Figure 1C), whereas the N-banding results revealed no satellite structure (Figure 1D). We subsequently employed CNV-seq technology to confirm the patient’s genotype. The CNV-seq results revealed seq[hg19]dup(18)(p11.32p11.21)chr18:g.120000_14980000dup, revealing a 14.86 Mb mosaic duplication (copy number 3.5) at 18p11.32p11.21. This finding confirms the presence of mosaic partial tetrasomy of the short arm of chromosome 18 (Figure 2). Consequently, the chromosomal karyotype was definitively determined as 47,XX,i(18)(p10)[85]/46,XX[15]. To investigate the origin of the patient’s abnormal chromosomes, we performed family studies and genetic testing. The chromosomal karyotypes of the patient’s parents and three younger sisters were all normal, indicating a *de novo* mutation (Table 1).

**Table 1:** Summary of phenotypic characteristics of cases with tetrasomy 18p compared to our case.

Patient	Age	Gender	Clinical characteristics
Our case	17 years	Female	Facial, dysmorphism, low hairline, strabismus, small and low-set ears, an absence of fingerprints, curved index fingers, and intellectual disability.
Case 1 (13)	4 years		Microcephaly, frontal bossing, hypertelorism, upslanting palpebral fissures, bilateral epicanthal folds, intermittent esotropia and bilateral mild myopia, bilateral ear pits, low-set ears, and a depressed nasal bridge.
Case 2 (14)	2.9 years	Male	Small head, protruding forehead, flat the bridge of the nose low, low ear, protruding tragus, flat pillow and widened distance between toes in the shape of “grass treading feet,” a right hydrocele of the external genitalia, and slightly high muscular tension of limbs leading to uncoordinated movement.
Case 3 (15)	0.7 years	Male	Microcephaly, growth delay, hypotonia, and cerebellar and renal malformations.
Case 4 (16)	2.1 years	Female	Microcephaly, mild generalized spasticity, arched eyebrows, horizontal palpebral fissures with unilateral convergent strabismus, bilateral epicanthic folds, small nose, well placed ears, oral cavity with high arched palate and upper vestibular frenula, tented mouth with slightly everted upper lip, hands with normal palmar creases and long fingers.
Case 5 (17)	1.2 years	Female	Relative micrognathia. Mild synophrys, long, curly eyelashes, antimongoloid slant, and bilateral internal strabismus. The ears were low set and the nose was pinched up. The mouth was rather small with a long philtrum, asymmetrical lips on crying, high arched palate, a shift of the uvula to the left, clinodactyly of the fifth fingers bilaterally, tapering fingers, asymmetrical length of the legs, and muscular hypertonia.

## 4 Discussion

Tetrasomy 18p syndrome is a rare chromosomal abnormality characterized by the presence of an additional marker chromosome consisting of an isochromosome of the short arm of chromosome 18. This structural anomaly is typically undetectable or uncharacterizable via conventional chromosome banding techniques and requires molecular genetic methods for accurate analysis. The size of this marker chromosome is equal to or smaller than that of chromosome 20 in the same metaphase spread (3, 4). The incidence rate is approximately 1/140,000 to 1/180,000 (1, 2). Its primary clinical features include facial deformities, neurodevelopmental disorders, growth abnormalities, and skeletal malformations. The pathogenesis of 18p tetrasomy syndrome is complex, with the most likely mechanisms being maternal meiosis II nondisjunction, centromere misdivision, or isochromosome formation (5). Small marker chromosomes are prone to loss, often resulting in mosaicism. O’Donnell et al. suggested that the development of this condition may be associated with advanced maternal age and oocyte aging (6). In this study, since the patient’s appearance showed significant differences from that of normal individuals, we considered the possibility of chromosomal abnormalities and therefore recommended chromosomal testing during the initial diagnosis. Abnormalities were detected during G-banding chromosomal analysis, prompting us to perform C-banding and N-banding karyotype analyses. The patient’s G-, C-, and N-banding karyotype results were 47,XX,+mar[85]/46,XX[15], indicating mosaicism with an additional small marker chromosome containing a centromere but lacking satellites and heterochromatin. However, the composition of the marker chromosome could not be definitively identified, nor could its pathogenicity be assessed. To further investigate its etiology, we conducted CNV-seq testing. Further validation via CNV-seq technology revealed a mosaic duplication of 14.86 Mb in the 18p11.32--p11.21 region (copy number: 3.5). By integrating cytogenetic and molecular techniques, the marker chromosome composition and breakpoint were clarified, ultimately confirming the karyotype as 47,XX,+i(18)(p10)[85]/46,XX[15]. Although G-banding karyotype analysis remains the “gold standard” for diagnosing chromosomal disorders—enabling comprehensive identification of whole-genome numerical and structural abnormalities and serving as the gold standard for balanced rearrangements—this method has inherent limitations. It offers limited resolution for detecting microdeletions and microduplications, relies on cell culture, and cannot be used to precisely determine the origin or size of marker chromosomes (7). C-banding primarily aids in identifying the origin of marker chromosomes, such as isochromosomes and monocentric or dicentric chromosomes, as well as in conducting polymorphism analysis. N-banding helps eliminate false positives arising from rDNA. The CNV-seq technique offers high resolution, whole-genome coverage, independence from cell culture, high-throughput automation, and high sensitivity for detecting mosaicism. Compared with chromosomal karyotype analysis, CNV-seq has distinct advantages in detecting small chromosomal deletions and duplications. It can accurately identify chromosomal fragments, regions, and sizes of unknown origin. However, this technique cannot detect balanced rearrangements or precisely determine chromosomal karyotype morphology distribution (8). This study identified discrepancies between the two detection techniques, as evidenced by inconsistent mosaic ratios and band localization patterns, which may be attributed to the inherent limitations of each method. N-banding revealed no satellite presence, effectively ruling out satellite fusion of the D and G group chromosomes. C-banding revealed no heterochromatin in the marker chromosome, which displayed a distinct centromere. On the basis of the centromere position and short-arm symmetry, the chromosome was identified as i(18p), a result that demonstrated greater diagnostic reliability. These findings highlight the significant diagnostic value of both C- and N-banding techniques for marker chromosome identification. CNV-seq technology cannot detect centromeric and heterochromatic regions; it can identify only duplications at breakpoint p11.32–p11.21. CNV-seq analyzes interphase cells and is unaffected by the cell culture process, allowing for a more accurate representation of the true chromosomal abnormalities in the sample. In contrast, chromosomal karyotype analysis is influenced by factors such as selective cell growth during culture, slide preparation, and random counting, which may lead to discrepancies in mosaic ratios (9). Therefore, for such marker chromosomes, a combination of G-banding, C-banding, and N-banding karyotype analyses with CNV-seq technology should be employed to improve diagnostic accuracy.

In this study, CNV-seq analysis revealed mosaic duplication of a 14.86 Mb region (copy number 3.5) on chromosome 18p11.32--p11.21, encompassing 58 protein-coding genes, which were classified as pathogenic variants. This finding correlates with clinical manifestations, including developmental delay, intellectual disability, craniofacial abnormalities, and ventricular septal defects. Giordano et al. reported a familial case involving two individuals carrying a duplication of 18p11.31-p11.23. The proband presented the following primary clinical manifestations: short stature, developmental delay, intellectual disability, moderate psychomotor retardation, microcephaly, and distinctive facial features, including a triangular face, high forehead, low-set ears, micrognathia, epicanthal folds, and strabismus. Additional findings included pes planus, cerebellar vermis hypoplasia, chorioretinal coloboma, growth hormone deficiency, hearing loss, fetal distress, intrauterine growth restriction, pneumothorax, and dilation of the lateral and third ventricles. The proband’s mother presented with partial hearing loss and mild micrognathia (10). Balasubramanian et al. reported a family with three cases of 18p11.32-p11.31 duplication. The elder sister presented primary clinical manifestations, including relative microcephaly, delayed language development, delayed fine motor skills, learning disabilities, and behavioral disorders. The younger sister primarily presented with cognitive impairment and behavioral disorders, whereas the father presented with learning disabilities and behavioral disorders (11). Uwineza et al. reported a case involving a 6-year-old girl with atrioventricular and atrial septal defects. Genetic analysis revealed a 4 Mb duplication in the 18p11.32-p11.21 region and a 2,925 kb duplication in the 18p11.21-q11.2 region, whereas the copy number in the approximately 1.1 Mb region of 18p (14,241,744–15,345,079) appeared normal (12). The patient exhibited clinical features, including a flat facial profile, low-set ears, downward-slanting palpebral fissures, and a small mouth. Consistent with the literature cited above, the patient in our study presented with mosaic partial tetrasomy of 18p (47,XX,+i(18)(p10)[85]/46,XX[15]). The clinical manifestations primarily included short stature, a low hairline, strabismus, small and low-set ears, an absence of fingerprints on the palms, curved index fingers, and intellectual disability. The patient was able to engage in simple communication, perform household chores, and maintain basic self-care but was unable to read, write, or perform calculations. Routine ultrasound examination revealed a slightly small uterus. The patient was capable of simple communication, performing household chores, and maintaining self-care, which may be attributed to the patient’s mosaic condition. Compared with previously reported cases involving abnormalities in the short arm of chromosome 18, our case presents a novel finding: the patient presented adermatoglyphia (absence of fingerprints). This observation provides additional clinical data that enhance the understanding of this disease category. The case of a teenage female with mosaic tetrasomy 18p is exceptionally rare worldwide. Phenotypic variability depends on the proportion and distribution of abnormal cells. Since the parental chromosomes showed no abnormalities, this case was considered a case of *de novo* variation. The patient’s parents, concerned about their daughter’s irregular menstrual cycles and the potential impact on her health and fertility, requested medical treatment with the expectation of restoring regular menstruation. Treatment with progesterone capsules (100 mg twice daily) was initiated to induce menstruation, with unsatisfactory results. From June 2025, the patient received Complex Packing Estradiol Tablets/Estradiol and Dydrogesterone Tablets 1 mg once daily combined with progesterone capsules (100 mg twice daily), which resulted in regular menstrual cycles. Currently, the patient has regular menstrual cycles, but the impact on fertility remains unknown and requires longer-term follow-up for confirmation. The patient’s karyotype is 47, XX, +i(18)(p10) [85]/46, XX [15] (10). However, genetic risks persist for the offspring of such patients. Preimplantation genetic testing (PGT) or prenatal diagnosis following natural conception is recommended to prevent the birth of affected children.

Although we have described the manifestations, diagnosis, and treatment of patients with Mosaic Partial Tetrasomy 18p, the experience from a single case is insufficient to represent the complete spectrum of this disorder or to ensure the generalizability of these findings to broader patient populations. Furthermore, due to the relatively short follow-up period, we were unable to comprehensively assess patients’ long-term clinical progression, late complications, or the sustained efficacy of any interventions. These factors collectively limit the strength of the observational associations in this study and preclude more definitive therapeutic conclusions. Therefore, additional cases are needed to support these findings.

## 5 Conclusion

In our study, we identified the etiology and genetic characteristics of a case involving mosaic partial tetrasomy of the short arm of chromosome 18 through clinical and genetic analysis. This case represents the first reported instance of clinical features in a patient with mosaic partial tetrasomy 18p. The absence of fingerprints on the palms and oligomenorrhea may represent novel characteristics of this condition. For such rare chromosomal abnormalities, the combined use of G-banding, C-banding karyotype analysis, CNV-seq technology, and comprehensive clinical evaluation can facilitate early diagnosis, genetic counseling, and personalized treatment.

## Data Availability

The raw data supporting the conclusions of this article will be made available by the authors, without undue reservation.
