# Correction: A rare case of mosaic partial tetrasomy 18p presenting with oligomenorrhea and intellectual disability: a case report

**DOI:** 10.3389/fmed.2026.1803076

**Published:** 2026-02-24

**Authors:** Guosheng Deng, Weiwu Liu, Yuqing Lai, Jinjie Pan, Yanyan Chen, Jujie Song, Donghua Zhang, Xiafei Liang, Sisi Ning, Yinghong Lu, Yunning Liang, Derong Li

**Affiliations:** 1 Clinical Laboratory, Yulin Maternal and Child Health Care Hospital, Yulin, Guangxi, China; 2 Obstetrics Outpatient Clinic, Yulin Maternal and Child Healthcare Hospital, Yulin, Guangxi, China; 3 Surgical Department, Yulin Maternal and Child Health Care Hospital, Yulin, Guangxi, China; 4 Reproductive Medicine Center, Yulin Maternal and Child Health Care Hospital, Yulin, Guangxi, China

**Keywords:** chromosome, chromosome abnormality, CNV-seq, oligomenorrhea, tetrasomy 18p

There was a mistake in the **Discussion**, Paragraph two. Duplicate content was identified as follows:

The patient's parents, concerned about their daughter's irregular menstrual cycles and the potential impact on her health and fertility, requested medical treatment with the expectation of restoring regular menstruation. Treatment with progesterone capsules (100 mg twice daily) was initiated to induce menstruation, with unsatisfactory results. From June 2025, the patient received Complex Packing Estradiol Tablets/Estradiol and Dydrogesterone Tablets 1 mg once daily combined with progesterone capsules (100 mg twice daily), which resulted in regular menstrual cycles. The patient's parents, concerned about their daughter's irregular menstrual cycles and the potential impact on her health and fertility, requested medical treatment with the expectation of restoring regular menstruation. Treatment with progesterone capsules (100 mg twice daily) was initiated to induce menstruation, with unsatisfactory results. From June 2025, the patient received Complex Packing Estradiol Tablets/Estradiol and Dydrogesterone Tablets 1 mg once daily combined with progesterone capsules (100 mg twice daily), which resulted in regular menstrual cycles.

A correction has been made to the section **Discussion**, Paragraph two:

“The patient's parents, concerned about their daughter's irregular menstrual cycles and the potential impact on her health and fertility, requested medical treatment with the expectation of restoring regular menstruation. Treatment with progesterone capsules (100 mg twice daily) was initiated to induce menstruation, with unsatisfactory results. From June 2025, the patient received Complex Packing Estradiol Tablets/Estradiol and Dydrogesterone Tablets 1 mg once daily combined with progesterone capsules (100 mg twice daily), which resulted in regular menstrual cycles.”

There was a mistake in the **Discussion**, Paragraph two. The number “[15]” was missing from the following: 47, XX, +i(18)(p10) [85]/46, XX.

A correction has been made to the section **Discussion**, Paragraph two so it now reads as follows: 47, XX, +i(18)(p10) [85]/46, XX [15].

The original version of this article has been updated.

